# Bridging the Gap between Genotype and Phenotype via Network Approaches

**DOI:** 10.3389/fgene.2012.00227

**Published:** 2013-05-31

**Authors:** Yoo-Ah Kim, Teresa M. Przytycka

**Affiliations:** ^1^National Center for Biotechnology Information, National Institutes of Health, National Library of MedicineBethesda, MD, USA

**Keywords:** networks, genotype-phenotype relation, information flow, gene expression, complex, complex diseases, cancer

## Abstract

In the last few years we have witnessed tremendous progress in detecting associations between genetic variations and complex traits. While genome-wide association studies have been able to discover genomic regions that may influence many common human diseases, these discoveries created an urgent need for methods that extend the knowledge of genotype-phenotype relationships to the level of the molecular mechanisms behind them. To address this emerging need, computational approaches increasingly utilize a pathway-centric perspective. These new methods often utilize known or predicted interactions between genes and/or gene products. In this review, we survey recently developed network based methods that attempt to bridge the genotype-phenotype gap. We note that although these methods help narrow the gap between genotype and phenotype relationships, these approaches alone cannot provide the precise details of underlying mechanisms and current research is still far from closing the gap.

## Introduction

The rapidly decreasing cost of genome-wide profiling and whole-genome sequencing stimulated an enormous amount of progress in mapping complex traits in humans and model organisms (Stranger et al., 2011). As of 2011, the NHGRI Catalog of Published Genome-Wide Association Studies (www.genome.gov/gwastudies) contained data from more than a thousand GWAS publications. However uncovering genotype-phenotype association is only the first step and such associations do not typically provide the explanation of the molecular mechanism behind the relationship. In addition, identified associations explain only a limited amount of heritability (Visscher et al., 2008; Witte, 2010), suggesting that the picture is far from complete at the level of association identification. The potential impact of rare variants (Visscher et al., 2008; Cirulli and Goldstein, 2010) and epistatic interactions (Cordell, 2009) complicates the inference of the underlying mechanisms even further. Indeed, in complex diseases various combinations of genomic perturbations often lead to the same organismal level phenotype. Therefore many of complex diseases are now commonly thought of as diseases of pathways (Califano et al., 2012). In the context of the above mentioned challenges, a pathway-centric perspective is fundamental to the understanding of the mechanisms of complex diseases and the identification of potential drug targets. However, this view exposes several computational and algorithmic challenges including (i) how to identify such dysregulated pathways, (ii) how to connect them to the genetic causes, and (iii) how to leverage the pathway-centric view to capture differences between different disease subtypes.

**Figure 1:**
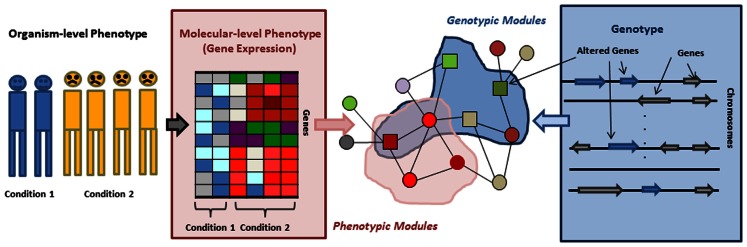
**Organismal/molecular phenotype, genotype, and genotypic/phenotypic modules**. Phenotypic modules can be identified by overlaying gene expression on an interaction network and searching for subnetworks/modules in which genes are differentially expressed or interactions are perturbed. Mapping genes residing in altered genomic regions to an interaction network allows for detecting genotypic modules.

In this review we survey the recent progress in network based approaches to address the above challenges. Many of these approaches start by replacing the organismal level phenotype, such as a disease, with molecular level phenotypes, such as gene expression. Thus we start by describing approaches that uncover the relation between organismal level phenotypes and molecular, network level phenotypes. Genes whose expression is often perturbed in concert with perturbation of an organismal level phenotype are not uniformly distributed in the network but rather form *phenotypic modules* (Figure 1). Thus, we subsequently describe network based approaches focused on identification of such phenotypic modules, their roles in different disease subtypes, and their ability to explain the heterogeneity of complex diseases. Next we switch from the phenotype-centric point of view to a more genotype centric perspective. It has been observed that genes that have aberrations associated with a given disease tend to belong non-randomly to subnetworks of the interaction network, which we refer to as *genotypic modules*. We then describe new algorithms to identify such modules. Finally, we discuss the approaches that combine these genotypic and phenotypic centered view-points and use molecular networks to model information flow from a genotype to correlated molecular phenotype, attempting in this way to bridge the gap between them. We conclude the review with a discussion of the power and limitations of the current approaches.

## Phenotypic Modules

Organismal level phenotypes such as diseases are always related to some molecular level changes, the so called molecular phenotypes. These include, for example, the over- or under expression of particular genes (Figure 1). Therefore one of the first steps toward understanding how organismal level phenotypic variants arise is to identify the molecular level phenotypes that accompany them. In the last decade gene expression emerged as a molecular level trait that can ultimately be used as such a molecular phenotype and be utilized for disease classification, identifying drug targets, and inferring interactions between genes. Systematically analyzing gene expression changes in different conditions and in the context of their molecular interactions usually leads to more robust and easier to interpret results than focusing on individual genes. Moreover, we do not know the function of most genes and, even when the function is known, many genes are pleiotropic and their function can only be interpreted in a context dependent way. Therefore recent methods, building on the observation that a molecular perturbation typically affects whole modules and not just individual genes, focus on identifying *phenotypic modules* – clusters of genes or pathways – significantly enriched with genes whose expression changes are correlated with phenotypic changes. An additional benefit of a module based approach is that the increased statistical power allows the identification a perturbed module even if the perturbation of each individual gene in the module might not be statistically significant. Finally, most phenotypes are complex and can emerge in many different ways. Thus, although we eventually would like to understand the subtle differences among individuals, the first line of attack is to capture the molecular pathways whose dysregulation is common across various disease cases.

### Identifying phenotype related genes and modules

One of the first network based methods to capture the impact of perturbation experiments on a gene network was proposed in the work of Ideker et al. (2002). Aiming to identify regulatory and signaling pathways, they integrated yeast protein–protein and protein-DNA interactions with gene expression changes measured in response to perturbations of the yeast galactose utilization pathway. Then they used simulated annealing to search for “*active subnetworks*” – sets of connected genes with significantly differential expression (Figure 1). Using this algorithm (jActiveModules, available as a cytoscape plugin), they were able to identify several subnetworks enriched with well-known regulatory and signaling pathways. This study provided a proof of concept for subsequent network based approaches. Compared to clustering methods based exclusively on gene expression data, one of the benefits of integrative network based approaches is that subnetworks identified by such methods can include genes that are not necessarily differentially expressed but still play an important role within a module by mediating a connection between genes with significant expression changes. For example, they were able to identify several genes connected by a common transcription factor, which only shows moderate changes in its gene expression level and thus would have been difficult to identify without context dependent methods.

The “active subnetworks” approach identifies modules containing differentially expressed genes without otherwise quantifying the relationships between the genes or their expression. However similarity between expression patterns may be important to identify functional modules. For example, if the expression changes of two neighboring nodes are correlated with each other, this might suggest that the two genes have related functional roles. To utilize this information, Ulitsky et al. (2010) developed the MATISSE algorithm to identify *Jointly Active Connected Subnetworks* (JACS) which are connected subnetworks with high average internal expression similarity (Ulitsky and Shamir, 2007). Computing the weight between each pair of genes based on expression similarity (e.g., the Pearson correlation) and gene specific confidence level that a gene is transcriptionally regulated under a given condition, they identified a set of connected genes with heavy weight in the osmotic shock response network in yeast and the human cell cycle network. A variant of this approach was subsequently used to identify regulatory networks defining phenotypic classes of human cell lines (Müller et al., 2008).

Analyzing subnetwork expression pattern also proved helpful for predicting genes contributing to the emergence of cancer. The IDEA (Interactome Dysregulation Enrichment Analysis) method is one such approach introduced by Mani et al. (2008). Unlike approaches that identify perturbed subnetworks by looking at dysregulated nodes, the IDEA method focuses on the identification of perturbed network edges. Specifically, using a combined interaction network [PPI, transitional, signaling, posttranslational modifications predicted by Modulator Inference by Network Dynamics (MINDy); Wang et al., 2006] as the underlying network, they searched for the edges connecting genes which in the disease state show loss or gain of expression correlation. They stipulated that genes enriched with adjacency to such perturbed edges are likely to play important roles in cancer and in this way identified several cancer related genes. Using this approach, they identified BCL2 as the gene adjacent to the largest number of dysregulated edges in FL lymphoma. This analysis also identified the SMAD1 gene, which could not be detected by differential expression analysis. Analysis of other cancer types also supported the utility of the method. Notably, MINDy (Wang et al., 2006), the posttranslational modification prediction algorithm used in that study as one of the sources for constructing the underlying network, provides an important step toward addressing another challenge in network analysis. Namely, MINDy tests whether the conditional mutual information, between a transcriptional factor TF and a target *t*, is non-constant as a function of a modulator *M*. In that case, *M* is inferred as a candidate posttranslational modulator of the TF. This approach has been subsequently used to produce the first genome-wide map of the interface between signaling and transcriptional regulatory programs in human B cells (Wang et al., 2009).

Another challenge that only recently started to be addressed is the issue of tissue specificity and cell-to-cell communication. Tissue specific gene expression can be used to understand the tissue specificity of networks. In a recent study, Keller et al. (2008) analyzed gene expression data in six different mouse tissues from an obesity-induced diabetes-resistant and a diabetes-susceptible strain before and after the onset of diabetes, and identified co-expression modules within and between tissues. The emergence of the between-tissue modules provides evidence for intercellular communication. In addition, they found that the cell cycle regulatory module in islets predicts diabetes susceptibility.

### Classification based on phenotypic modules

Differentially expressed modules have been successfully used for disease classification (Tan et al., 1996; Ideker et al., 2002; Chuang et al., 2007; Lee et al., 2008a; Dao et al., 2010). In their pioneering work, Chuang et al. (2007) utilized protein–protein interaction networks to improve the classification power of metastasis in breast cancer. Specifically, they identified connected subnetworks in which the expression patterns of genes significantly differ between the two cancer types. To select such subnetworks, they first defined network activity score based on the aggregate value of a differential expression measure of all genes in the subnetwork. Comparing the vectors of activity scores between samples of different types (metastatic or non-metastatic) allowed them to identify subnetworks whose activity discriminates the two cancer types. They searched for subnetworks with high discriminative power in a greedy manner. Importantly, the identified subnetworks can be considered to be potential markers. As in the case of single gene disease markers, a network marker will distinguish some but not all disease cases and multiple subnetworks might be necessary.

The approach of Chuang et al. (2007) provided the proof of principle for the utility of network based methods in disease classification and stimulated further research in this direction. Other approaches suggested later differ mostly in how the candidate network markers are identified and how the final set of classifying subnetworks is selected from this candidate set. For example, instead of protein–protein interaction network, Lee et al. (2008a) utilized curated path ways as the underlying network.

More recently, Dao et al. (2010, 2011) developed an alternative network based approach for the classification of cancer subtypes. They utilized an edge weighted PPI network based on the confidence score of each interaction, and searched subnetworks with sufficient edge weights (Dao et al., 2010). They additionally required all genes in a network marker to be consistently differentially expressed in a certain minimal number of samples. Their subsequent improvement included a more advanced, graph color coding based algorithmic approach for selecting optimally discriminative set network markers (Dao et al., 2011). Using it to predict drug responses to cancer treatment, they found that the algorithm not only provided better and more stable predictive power but also was able to obtain more reproducible markers compared to the previous methods.

In a different study, Chowdhury and Koyuturk (2010) developed a set cover based algorithm (see also subsection Disease Heterogeneity and Network Cover) for the purpose of cancer classification, and in a follow-up study they used the mutual information between the gene expression levels and disease phenotypes to measure how informative a subnetwork is for classification. To select the most informative subnetwork markers they used a bottom-up enumeration approach to exhaustively search all possible subnetworks.

### Disease heterogeneity and network cover

Most observed organism-level phenotypes arise in a heterogeneous way. Diseases such as autism, cancer, or diabetes are now seen as a spectrum of related disorders that manifest themselves in a similar fashion. Despite the differences, such disorders are expected to share some common molecular level features whose identification should be helpful for understanding the disease. Set cover approaches have been found to be useful in capturing heterogeneity among patients in complex diseases (Chowdhury and Koyuturk, 2010; Ulitsky et al., 2010; Kim et al., 2011a). In these approaches a gene is considered to cover a disease sample if it is differentially expressed in the sample. Given gene expression profiles, a set cover method selects a subset of genes, so that each gene is covering a group of patient samples and so that the genes in the selected set also satisfy other conditions including, for example, minimization of the number of selected genes. The main idea is that selected genes will collectively represent the heterogeneous disease cases. Building on this intuition and aiming to detect dysregulated pathways in complex diseases, Ulitsky et al. (2010) extended the set cover technique by integrating expression data and interaction networks. Their method, named DEGAS (*de novo* discovery of dysregulated pathways) searches for a smallest set of genes forming a connected subnetwork so that each disease sample is covered by certain minimal number of genes from this set. This way they find a connected subnetwork collectively covering all the disease samples. They utilized this algorithm to identify significantly differentially expressed subnetworks in Huntington disease as well as breast cancer studies. Finally, Chowdhury et al. (2011) and Chowdhury and Koyuturk (2010) developed a network cover based algorithm for disease/control classification. The algorithm starts from a node and greedily extends the subnetwork to find the smallest connected set of genes (called “coordinately dysregulated subnetwork”) that are collectively and consistently differentially expressed in (thus covering) all disease samples. Among the subnetworks for all seed genes, they selected the markers of the most discriminative potential based on mutual information.

## Genotypic Modules

**Figure 2:**
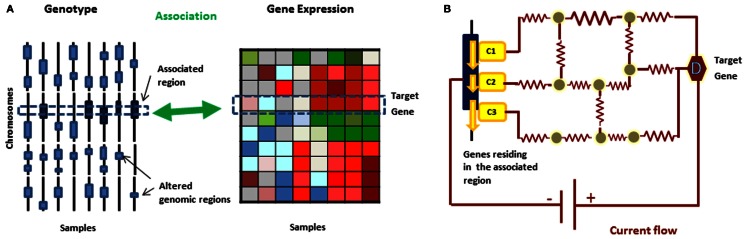
**(A)** In eQTL analysis, gene expression is treated as a quantitative phenotype and genetic loci controlling the phenotypic changes can be identified based on correlations between the genomic variations and expression profiles from the same set of samples. **(B)** A current flow network algorithm can be used to prioritize the candidate disease causing genes in the genomic region and uncover molecular mechanism behind the relationship simultaneously.

In the previous section, we discussed approaches that identify phenotypic modules – subnetworks whose expression changes are correlated with phenotypic changes. In this section, we turn our attention to the genetic causes of perturbations and subnetworks defined by these causes. Recent studies suggested that genomic alterations in complex diseases, such as cancer and neurological disorders, are significantly heterogeneous. However, it has been proposed that the mutated or altered genes may belong to the same pathways, collectively dysregulating these pathways. For example recent large scale studies in sporadic autism showed that 39% (49 of 126) of the most severe or disruptive *de novo* mutations map to a highly interconnected β-catenin/chromatin remodeling protein network (O’Roak et al., 2012). This hypothesis has led to the emergence of approaches to detect disease associated pathways (Bergholdt et al., 2007; Gilman et al., 2011; Rossin et al., 2011; Vandin et al., 2011) which focus on identification of *genotypic modules* – subnetworks that are enriched with genes having disease associated genetic alterations (Figure 2). In the case of cancer, this methodology is typically applied to the somatic cell mutations which are the most direct triggers of the disease.

Typically, searching for genotypic modules starts with the identification of genomic regions that are frequently altered in a disease of interest and mapping the genes residing in the altered regions to a network. Next, modules enriched with the genetically altered genes are identified. Genotypic modules are defined based on network topology and possibly other information such as known functional relationships between the genes, but unlike phenotypic modules, they do not assume that molecular phenotype data such gene expression is available. Several different ways to score the modules utilizing their connectivity and similarity have been proposed. An important challenge in such approaches is to develop rigorous statistical tests to evaluate the significance of the subnetworks.

Vandin et al. (2011) introduced a computational framework, called “*HOTNET*,” to identify subnetwork in which genes are mutated in a significant number of patients. To this end, they measured the “influence” between two genes using a diffusion process (Qi et al., 2008) in a protein interaction network. Then they used this measure to construct a weighted “influence graph” between mutated genes. Then they identified a significant subnetwork of fixed size covering a maximum number of disease cases. Finally, they employed a rigorous two-stage multiple hypothesis testing correction method to control the false discovery rate (FDR) for the identified subnetworks. The method was applied to ovarian cancer analysis in TCGA (the cancer genome atlas) and identified the NOTCH signaling pathway which is indeed known to be significantly mutated in cancer samples (Bell et al., 2011).

The NETBAG (NETwork Based Analysis of Genetic associations) method is a related method that has been developed by Gilman et al. and applied to identify a biological subnetwork affected by rare *de novo* copy number variations (CNVs) in autism (Gilman et al., 2011; Levy et al., 2011). Due to their rarity, new (*de novo*) germline variations (as opposed to inherited variations) are often not statistically significant and require an integrated network based approach to understand their functional impacts. In the NETBAG method, a background network is constructed so that edges are assigned the likelihood odd ratio for contributing to the same genetic phenotype. The likelihoods were computed using a naïve Bayesian approach (similar to the method used to build functional networks; Lee et al., 2004, 2008b) based on various descriptors of protein function such as GO annotations, protein–protein interactions, sequence homology, etc. Genes with CNV were then mapped to the likelihood network and connected clusters of such genes were identified. A greedy growth algorithm was used to find the cluster with maximal score which was computed as direct multiplication of the likelihoods. The significance of a cluster score was estimated by the distribution of maximal scores for clusters obtained from randomized data. Applying the method to a rare *de novo* CNV dataset from Autism samples (Levy et al., 2011), they identified a CNV affected subnetwork, which is significantly enriched with synaptogenesis and axon guidance related GO terms.

Rossin et al. (2011) proposed another approach to identify genotypic modules, which is the basis for the DAPPLE (Disease Association Protein–Protein Link Evaluator) algorithm. They considered all proteins that are encoded by genes in the genomic region of interest and connected those proteins based on protein interaction data. They identified direct and indirect subnetworks: a direct subnetwork only consists of genes in the regions with genomic variants and direct interactions between them. In an indirect subnetwork, they allowed genes to be connected via common interactors, therefore being at most two hop neighbors in the protein interaction network. To evaluate if the resulting subnetwork has properties different from a random subnetwork they computed subnetwork scores based on several variants of connectivities, i.e., the number of edges in the network or the average degrees of common interactors. The significance of the network was estimated via a permutation test where random networks are generated by shuffling node labels among the same degree nodes. Applying the method to the genomic regions known to be associated with Rheumatoid Arthritis (RA) and Crohn’s disease (CD) from previous GWAS studies, they found that the identified subnetworks have significantly more connected. Scoring individual genes based on their connectivity scores and the permutation method, they further proceeded to nominate high scoring genes from associated regions as candidates for influencing disease risk and found significant differences in the expression between the nominated genes and the remainder of genes.

## From Genotype to Phenotype

The approaches discussed in the previous sections dealt with modules of genes associated with either phenotypic or genotypic differences. While both are helpful for predicting dysregulated modules, a more effective way to understand disease mechanisms is by combining both genotypic and phenotypic data. A useful link between the two can be provided by expression quantitative trait loci (eQTL) analysis (Stranger et al., 2005, 2007) – a technique in which gene expression level is treated as a quantitative phenotype and genetic loci controlling the phenotypic changes are identified by comparing gene expression and genotypic data from the same set of samples and determining the associations between them. However eQTL analysis alone does not provide the underlying molecular mechanism through which the information on genetic alteration is propagated. Consequently several methods have been proposed to fill this gap.

### Disease associated modules using expression, genotype, and other data

One way to start bridging the gap between genotype and phenotype is to link genetic variations or genotypic modules to phenotypic modules. One simple approach is to identify disease associated phenotypic modules and identify the eQTL associations of the module members (Chen et al., 2008; Kang et al., 2012). Using the approach, Chen et al. (2008) elucidated modules that are perturbed by susceptibility loci that in turn lead to a disease. Specifically they started by constructing co-expression networks for liver and adipose tissues collected from a segregating mouse population in the B × H cross. They found that sub-networks were enriched for a number of biological processes such as insulin signaling, inflammation, muscle-related processes as well as with genes that are perturbed by specific genetic loci. They also established that one subnetwork, which was macrophage-enriched, was likely to have causal relationship with metabolic traits.

An important challenge in modeling genotype-gene expression relations is posed by the fact that the observed variations in expression might reflect a composite effect of many genetic variations. To model such joint transcriptional effects of copy number aberrations on target mRNA expression, Jörnsten et al. (2011) developed a computational framework, named EPoC (Endogenous Perturbation analysis of Cancer). Given two matrices, **ΔX** and **ΔY**, CNA (Copy Number Alteration), and mRNA profiles of disease samples, they represented the transcriptional effects as

ΔY=GΔX+Γ

where **G** = {*g_ij_*} indicates the effects of CNA of gene *j* to the transcription of gene *i*. The matrix **G** is obtained by solving the linear equations using a Lasso method. **G** can be seen as a CNA-driven network defining the transcriptional effects between genes. The optimal network size (number of non-zero entries controlled by the lasso penalty) is estimated by comparing network consistency in terms of Kendall’s W or by optimizing mRNA prediction. Once the size of the network is estimated, the final network is computed by repeating the estimation and validation process via pseudo-bootstrapping and retaining interactions appearing with at least 20% frequency. Applying the method to glioblastoma data in TCGA, they not only found that some nodes emerging as network hubs are oncogenes and tumor suppressors with frequent copy number alterations, but also identified several other genes not previously known to be associated with glioblastoma but whose casualty to the disease is consistent with other evidence. Subsequently, they obtained prognostic scores using Singular Value Decomposition (SVD) of the network and showed that the scores successfully predict the survival time of patients whereas the transcriptional network or standard SVD from either mRNA or CNA profiles alone fails to predict patient survival effectively.

Several groups proposed alternative methods to identify co-expressed groups of genes and regulating loci at the same time. For example, Zhang et al. (2010) proposed a method based on a Bayesian partitioning approach where they used a Markov chain Monte Carlo (MCMC) strategy to identify groups of genes and their regulating loci simultaneously.

Another promising technique that allows for identifying modules together with their regulators has been pioneered by Segal et al. (2003). The goal of their approach is to identify coherently expressed modules and their regulatory programs. A regulatory program has the form of a decision tree with regulators in decision nodes so that the states of the regulators on the path from the root to a leaf (a module) determine the expression of the genes in a module. The number of inferred regulators (the nodes in the decision tree modeling the regulatory program) is typically small since the method attempts to capture the most influential regulators for the whole module. The modules and their regulatory programs are obtained through an iterative refinement process. In their first related method, Segal et al. considered a predefined set of putative regulators including transcription factors. This method has been later extended to include regulatory genetic variations and disease phenotypes (Lee et al., 2006, 2009a; Chen et al., 2009; Akavia et al., 2010; Kreimer et al., 2012). In particular, in the CONEXIC algorithm, genetic alterations, such as CNVs or mutations, were included as possible regulators and were tested whether gene expression in a module is switched from normal to the level characteristic to the disease state (Akavia et al., 2010).

The above approaches constructed modules using expression and genomic profile without taking advantage of interdependence between the data. In contrast, Kim and Xing proposed a statistical framework called graph-guided fused lasso (GFlasso) for QTL(Quantitative Trait Locus) analysis to identify genetic variations associated with multiple correlated traits simultaneously (Kim and Xing, 2009). They first constructed a Quantitative Trait Network (QTN) where each node represents a trait and edges correspond to the correlations between traits. For example, in the case of organismal phenotype, the weight might be correlated with height. For molecular phenotypes such as gene expression, this correlation could mean correlation in gene expression. For a given trait vector **y**, and genotype matrix **X**, the linear regression model is formulated as

y=Xβ+∈

where β and ∈ are the regression coefficient and error vector, respectively.

When applied to association studies with multiple traits, the basic Lasso method computes the regression coefficients by adding the L_1_ norm of coefficients (lasso penalty) to the residual sum of squares, which removes weak associations and provides sparse associations. In GFLasso, an additional penalty term is further added to ensure that two highly correlated phenotypes have associations with the same genomic variations. Namely, the penalty was added when two correlated traits have differences in regression coefficients, which presumably increases the power of detecting causal genomic variants to correlated traits. In this way, GFLasso associates traits with genotypic variations so that related traits are mapped preferentially to the same genotypic variations. That is, for a connected group of co-expressed genes a preference will be given to associations of these genes with a common genetic variation.

### Identifying causal genes and pathways using information flow

Although the approaches discussed above connected genotypic variation with phenotypic data, only few attempted to uncover intermediate genes that might mediate this relationship. For example, in the CONEXIC method mentioned above, transcription factors were identified as intermediate regulatory genes to complement genetic variations in the decision tree (Akavia et al., 2010). However, can a longer sequence of information flow be identified? To address this question, Zhu et al. (2008) combined multiple types of molecular data, including genotypic variations, expression variations, transcription factor binding, and physical interaction data and reconstructed a causal network. In short, Bayesian networks are directed acyclic graphs, where edges are defined by the conditional probability that represents the state of a node when the states of its parents are given. The reconstruction algorithm takes genetic data as the source of perturbation. Protein–protein interactions together with transcription factor binding data were used as prior evidence of a regulatory relationship. Specifically, protein interaction data was utilized to identify complexes that are co-regulated by a given transcription factor(s). To evaluate the results, the authors compared the set of the genes that could be reached from putative regulators in the genetic loci following directed links with the set of genes associated with the given loci in eQTL analysis. The intersection was significant in most cases, providing a proof of principle that such causal networks can provide cues on information propagation from genotype to phenotype.

An alternative approach is to utilize information flow where one can consider genotypic variation as the “source” of perturbation and genes with phenotypic changes as the target of a perturbation pathway. Information flow in the biological network has been used in previous studies for predicting protein functions, prioritizing candidate disease genes, and finding network centralities (Nabieva et al., 2005; Newman, 2005; Tu et al., 2006; Stojmirovic and Yu, 2007; Köhler et al., 2008; Suthram et al., 2008; Zotenko et al., 2008; Lee et al., 2009b; Missiuro et al., 2009; Yeger-Lotem et al., 2009; Vanunu and Sharan, 2010). In particular, a flow based approach can be used to augment network information to eQTL analysis, helping identify causal genes in genomic regions and understand the propagation of information signals from causal genes to their target genes. The simplest approaches would be to test if there is a path in the interaction network that connects a mutated gene to its putative target. The distance between the putative cause and target genes could be used to score the strength of the relationship. However, such approach would ignore the fact that the expression of all genes in all samples have known and thus could be used to guide the information flow. Specifically, we can use the expression data to assign weights to edges so that some edges are more likely to be used by the information flow than other.

We review here two different types of network flow approaches that can model such system – current flow network and minimum cost network flow. In current flow approaches, the network is modeled to mimic the behavior of current in an electronic circuit and a resistance is associated with each edge while network flow approaches resemble water finding paths through pipes and therefore associate capacities and weights with edges representing respectively the maximum amount of flow and the cost of sending flow through an edge. Both approaches have been successfully applied to uncover molecular mechanisms connecting two different types of data. It is worth noting, as pointed out below, that the current flow network provides an efficient framework equivalent to a random walk which is also often used for modeling information flow in biological networks.

In the context of connecting genetic perturbations to expression changes, Tu et al. (2006) proposed a random walk approach to infer causal genes and underlying causal paths over a molecular interaction network. They applied the method to the data obtained from yeast knock-out experiments. Given the expression profile of a target gene *g_t_* and an associated eQTL region, a number of random walks are repeatedly started from *g_t_* and the likelihood of a gene in the eQTL region to be causal is estimated by the number of times that the random walker arrives at the gene. Assuming that the activities of genes on a pathway are correlated with the expression level of the target gene, the weight of a gene *g* in the network is defined to be the absolute value of the Pearson’s correlation coefficient between the expression values of *g* and *g_t_*, and the transition probability of a random walk is computed based on the weights.

Using the analogy between random walks and current flow networks, Suthram et al. (2008) developed a method called eQED where they integrated eQTL analysis with molecular interaction information modeled as a current flow network. Specifically, each edge (*u*, *v*) is assigned the resistance that is inversely proportional to (|*corr*(*u*, *g_t_*)| + *|corr*(*v*, *g_t_*)|)/2 where *corr*(*x*, *y*) denotes Pearson’s correlation coefficient of the gene expression levels of gene *x* and *y*. They further considered the directions of links in molecular networks (e.g., TF-DNA interactions) and formulated the problem as a linear programming, for which the optimal solution can be efficiently computed.

We employed the circuit flow approach to identify causal genes and dysregulated pathways in Glioma, utilizing human interaction networks (Kim et al., 2011a,b). For a given target gene, an eQTL analysis typically finds multiple associated regions and simply applying a more stringent *p*-value cutoff may eliminate many true causal genes. Moreover, each region can contain dozens of candidate causal genes. Among these genes, we would like to identify the ones whose alterations are most likely to cause abnormal expression for a given target gene (Figure 2).

To identify potential causal genes in glioma we utilized CNVs in cancer tissues and gene expression profiles of the same set of patients. We first compared the gene expression levels in cancer patients to non-tumor cases and selected a set of differentially expressed genes as target genes using a set cover algorithm. Performing eQTL analysis, chromosomal regions where CNVs correlated with the gene expression changes were identified. Next we used the current flow algorithm to identify potential causal genes in the associated region. More specifically, for each selected target gene and an associated region, we created a circuit network where the target gene is a source of the current flow and the candidate genes residing in the region are included as the sinks. We computed the amount of current entering the candidate genes in the network and estimated an empirical *p*-value for each pair of a target and a causal gene, utilizing a permutation test, for which we ran the current flow algorithm for random networks, which we generated in a degree preserving way and all edges retained the same resistance.

Considering the genes that received a significant amount of current, we identified putative causal gene in Glioblastoma. In addition, by taking into account the amount of current going through intermediate nodes, we were also able to uncover commonly dysregulated pathways including Insulin Receptor signaling pathways and RAS signaling. Several hub nodes on the identified pathways such as EGFR were known to be important players in Glioma or more generally in cancer. Compared to simple genome-wide association studies which only identify putative associations between causal loci and target genes, the current flow based method provides increased power in predicting causal disease genes and uncovering dysregulated.

Yeger-Lotem et al. (2009) developed a minimum cost network flow based method named ResponseNet to uncover molecular mechanisms for responses to increased expression level of alpha-synuclein, a protein implicated in neurodegenerative disorders such as Parkinson’s disease. A minimum cost network flow is defined in a network with a source and a sink, and the goal is to minimize the total cost while sending flow from the source to the sink without violating the capacity constraints. To model the information propagation using a minimum cost network flow, Yeger-Lotem et al. (2009) first selected genetic hits which modify α-syn toxicity and connect them to the source of flow. Differently expressed genes are linked to the sink of network flow. The cost of an edge is computed based on the probability that the two endpoints interact in a response pathway, which is estimated based on experimental evidences. A constant negative cost is assigned to the links from the source. The capacity for a link from a target node to the sink is computed based on its transcript level while uniform capacity is assigned to all other links. Given the flow solution with minimum cost, a response network was predicted by ranking nodes in decreasing order of total incoming flows.

## Conclusion

The ever-new discoveries of associations between genetic variations and complex traits such as common human diseases, posed a key question – how can we close the gap in genotype-phenotype relationships. To answer this challenging question, a number of computational network based approaches have been developed as surveyed in this review. Focusing on groups of related genes leads to increased statistical power and enhances interpretability of the results. Through these method several new insights have been obtained including the involvement of macrophages in metabolic diseases (Chen et al., 2008) and the regulation of protein trafficking in melanoma (Akavia et al., 2010). Network and/or module based approaches also proved to be powerful in pinpointing disease causing genes, many of which, for example Ppm1l for metabolic syndrome or TBC1D16 and RAB27A for melanoma (Akavia et al., 2010), have been confirmed experimentally while others are supported by literature evidences.

One of the biggest challenges in understanding complex diseases relates to the fact that such diseases are highly heterogeneous. Therefore, in addition to being able to discover what individual disease cases have in common, we need to understand the differences between different disease subclasses. In this review, we have discussed several network based approaches for supervised disease classification.

**Figure 3:**
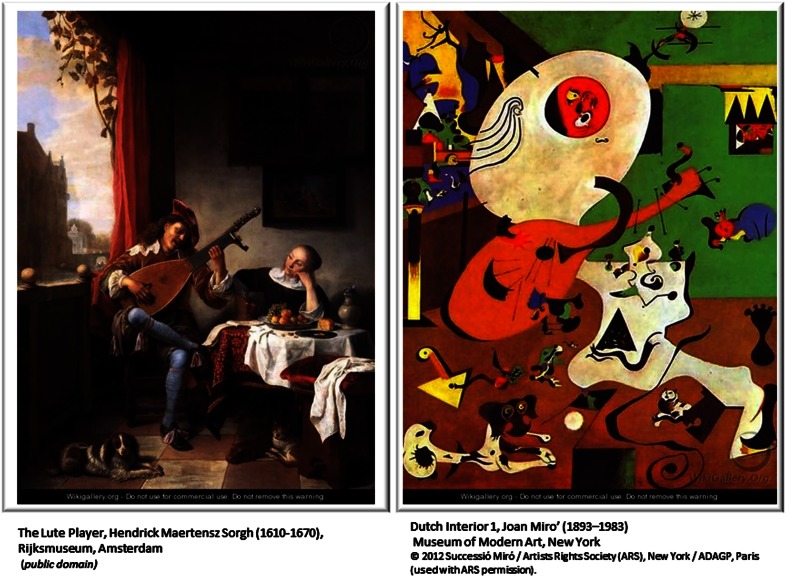
**Miró’s depiction of the Sorgh’s painting provides a good analogy for the relation between computationally inferred subnetworks and true biological pathways**. Such subnetworks provide somewhat distorted depiction of real relationships within the cell and while general components are distinguishable, much of the details are inaccurate. For example, Kim et al. (2011a) identified EGFR signaling as one of the subnetworks dysregulated in Glioma. However, if we compare the topology of the retrieved pathway with the topology inferred using laborious small scale experiments, we usually find that the topology of inferred subnetwork is distorted relative to the real pathway.

Finally, to fully understand a disease, we need to grasp the precise molecular mechanism behind it. The understanding of the mechanistic processes is ultimately necessary for guiding a rational design of drug therapies. While current network based approaches have certainly helped to understand the landscape of cellular level changes that accompany phenotypic changes, most of the results are of impressionist-type landscape, painted with the broad strokes of dysregulated pathways and groups of genes rather than with the precise and detailed molecular mechanisms. While the approaches that rely on physical interactions, such as the current flow approach, may be, in theory, the closest to explanatory details, they are also limited by incompleteness and inaccuracy of physical interaction data. Perhaps a good analogy is Miró’s interpretation of Sorgh’s painting “The Lute Player” (Figure 3). While some components (like the dog or the lute) are strong and clear despite some inaccuracy, others are less so. In fact much of what we can recognize or interpret in Miró’s painting depends on our knowledge of Sorgh’s original painting. This, in some sense, is also true for the interpretation of biological results obtained by computational network based approaches. They require some reference points such as GO categories, KEGG pathways, knowledge of a function of at least some genes, etc. for the interpretation of the results. For example, such a network based method could identify perturbation of known biological pathways such as EGFR signaling. However, if we compare the topology of such a pathway retrieved by these methods with “gold standard” knowledge obtained through many years of targeted, small scale experiments, we typically find that the topology of the inferred subnetwork is quite distorted relative to this gold standard.

These issues notwithstanding, current computational techniques with no doubt have made significant progress toward pinpointing commonly dysregulated pathways, disease classification, and identification of disease associated genes.

## Conflict of Interest Statement

The authors declare that the research was conducted in the absence of any commercial or financial relationships that could be construed as a potential conflict of interest.
